# Effect on Intraocular Pressure of Switching from Latanoprost and Travoprost Monotherapy to Timolol Fixed Combinations in Patients with Normal-Tension Glaucoma

**DOI:** 10.1155/2014/720385

**Published:** 2014-11-19

**Authors:** Ryoko Igarashi, Tetsuya Togano, Yuta Sakaue, Takaiko Yoshino, Jun Ueda, Takeo Fukuchi

**Affiliations:** Department of Ophthalmology and Visual Science, Niigata University Graduate School of Medical and Dental Sciences, 1-757 Asahimachi-dori, Chuo-ku, Niigata 951-8510, Japan

## Abstract

*Purpose*. To evaluate the effect on intraocular pressure (IOP) of switching from latanoprost and travoprost monotherapy to timolol fixed combinations in Japanese patients with normal-tension glaucoma (NTG). *Methods*. 27 NTG patients (54 eyes) were compared IOP, superficial punctuate keratitis (SPK) scores, and conjunctival injection scores in eyes treated with prostaglandin (PG) or PG analog/beta-blocker (PG/b) fixed-combination 6 months after the change in therapy. *Results*. The mean baseline intraocular pressure was 17.4 ± 1.59 mmHg in eyes receiving PG therapy only and 17.4 ± 1.69 mmHg in eyes switched to PG/b. Switching to fixed combination therapy from PG monotherapy, the mean IOP was 13.1 ± 1.79 mmHg (*P* < 0.001)  (−24.71% reduction from baseline) at 6 months. The mean conjunctival injection score was 0.69 for eyes on PG monotherapy and 0.56 for eyes on fixed combination therapy (*P* = 0.028). The mean SPK scores were 0.46 and 0.53. This difference was not statistically significant (*P* = 0.463). *Conclusions*. Switching from PG monotherapy to PG/b fixed combination therapy for NTG resulted in a greater intraocular pressure reduction than PG alone without increasing the number of instillations.

## 1. Introduction

Since timolol first entered the Japanese market as a topical medication for glaucoma in 1981, numerous other topical medications have been developed. In 1999, latanoprost was introduced as the first prostaglandin (PG) analog. Prostaglandin analogues have become the first-line treatment for normal-tension glaucoma (NTG) due to their ability to reduce intraocular pressure. However, when monotherapy does not sufficiently reduce intraocular pressure, beta-blockers and carbonic anhydrase inhibitors are selected [1]. In 2012, alpha-2 agonists and other agents were added to the list of options for treating glaucoma as a result of recent data indicating additional therapeutic effects such as neuroprotection [2, 3]; thus, the number of treatment options is increasing. In addition to reducing intraocular pressure, other factors should be considered when selecting agents, such as adverse effects, ease of administration, and indications to ensure the appropriate therapeutic strategy for each individual patient.

However, as the number of prescribed agents increases in a given patient, the risk of adverse effects also increases. According to a report by Fukuchi et al. [4], corneal epithelial disorders are observed in approximately 50% of patients using topical glaucoma medications. In particular, the incidence and severity of adverse effects increase as the number of instillations or formulations of eye drops increase.

One study investigated changes in the topical treatment protocol and long-term intraocular pressure in patients with broadly defined POAG before and after 1999, the year that latanoprost, dorzolamide, and timolol gel were introduced [5]. There was a statistically significant decrease in intraocular pressure in patients with both narrowly defined POAG and NTG. When comparing treatment regimens for POAG before and after 1999, the number of topical formulations prescribed per patient was nearly identical, but the number of instillations was significantly lower. For NTG, the number of prescribed formulations showed a statistically significant increase, while the number of instillations remained constant. Overall, intraocular pressure was reduced further and the treatment effect and efficacy were improved without an accompanying increase in the topical treatment burden.

There is now a deeper recognition of the importance of adherence and compliance in glaucoma therapy. The introduction of fixed combination therapy may help increase therapeutic efficacy [6]. Fixed combination therapy is advantageous over monotherapy in terms of patient adherence. One report demonstrated the safety and efficacy of transitioning from PG analog monotherapy to a PG analog/beta-blocker (PG/b) fixed-combination eye drop [7]. However, the majority of non-Japanese PG/b studies has been conducted on subjects with high intraocular pressure. Therefore, the present study examined the effects of fixed combination drops administered to Japanese NTG patients on the anterior chambers bilaterally.

## 2. Subjects and Methods

This study followed the tenets of the Declaration of Helsinki. All the patients' characteristics were shown in Table 1. A total of 27 NTG patients (54 eyes) undergoing follow-up care at Niigata University Medical and Dental Hospital and affiliated hospitals (Ojiya General Hospital and Niigata-Minami Hospital) were enrolled. The 27 subjects were comprised of 15 males and 12 females with a mean age of 58.1 ± 8.6 years. The inclusion criteria were as follows: intraocular pressure assessed pretreatment, NTG characterized by a ≤0.5 mmHg difference in intraocular pressure bilaterally without therapy, PG monotherapy in both eyes, and single-measure differences in intraocular pressure of ≤1 mmHg and a mean difference of ≤0.5 mmHg bilaterally. The PG analogs included latanoprost (Xalatan; 13 patients) and travoprost (Travatanz; 14 patients).

NTG was diagnosed according to the third edition of the Japan Glaucoma Society's Glaucoma Diagnosis Guidelines. Among patients with broadly defined POAG, NTG was defined as statistically prescribed normal intraocular pressure (20 mmHg) or lower in all intraocular pressure measurements during the course of progressive glaucomatous optic neuropathy.

Exclusion criteria consisted of the following: previous intraocular surgery; bronchial asthma; chronic obstructive pulmonary disease; heart failure; sinus bradycardia; atrioventricular block; cardiogenic shock. We exclude patients with active ocular inflammation and dry eye syndrome from this study (or patients receiving concomitant antihistamines, lubricants, or other eye drops during the study period). The protocol for the present study was approved by the Institutional Review Board of Niigata University Medical and Dental Hospital.

In each patient, the eye with the lower Humphrey 30-2 Program SITA standard MD value (poorer visual field) was switched from PG monotherapy to PG/b fixed combination therapy, while the eye with the higher MD value (better visual field) was continued on PG monotherapy. Intraocular pressure was measured bilaterally at 1, 3, and 6 months after the switch. Both eyes were then prescribed either PG monotherapy or PG/b fixed combination therapy, and the patients were reexamined 3 and 6 months later. Eye drops were instilled between 7:00 and 10:00 PM for all prescribed medications. All intraocular pressure measurements were performed between 9:00 AM and 12:00 PM (outpatient hours) using a Goldmann applanation tonometer and completed within 1 hour in all cases. The effect on corneal epithelial cells was determined by assessing the severity of diffuse superficial keratitis using the area-density (A-D) classification [8]. Superficial punctuate keratitis (SPK) scores were calculated as A × D as previously described. Conjunctival injection was graded as follows: easily observable conjunctival vessels (0 points); localized conjunctival redness (1 point); vivid conjunctival redness (2 points); evident conjunctival injection (3 points).

The following outcomes were compared: (1) intraocular pressure in eyes treated with PG or PG/b 6 months after the change in therapy; (2) SPK scores; (3) conjunctival injection scores. Changes in intraocular pressure were compared using the *t*-test. Conjunctival injection and SPK scores 6 months after the change in therapy were compared using the Wilcoxon signed-rank sum test. The PG data were compared using the Mann-Whitney *U* test. The level of statistical significance was set at *P* < 0.05.

## 3. Results

The mean baseline intraocular pressure (BL) was 17.4 ± 1.59 mmHg in eyes receiving PG therapy only and 17.4 ± 1.69 mmHg in eyes switched to PG/b. Immediately before starting PG/b, the mean intraocular pressure was 14.3 ± 1.71 mmHg in eyes continuing on PG and 14.2 ± 1.54 mmHg in eyes switched to a fixed combination. More specifically, BL was 17.6 ± 1.21 mmHg in eyes continuing on latanoprost and 17.6 ± 1.35 mmHg in eyes switched to latanoprost/timolol fixed combination (LTFC). BL was 17.4 ± 1.86 mmHg in eyes continuing on travoprost and 17.4 ± 2.00 mmHg in eyes switched to travoprost/timolol fixed combination (TTFC). The pretherapy intraocular pressure was 14.0 ± 1.41 mmHg in eyes continuing on latanoprost, 14.1 ± 1.37 mmHg in eyes switched to LTFC, 14.4 ± 1.86 mmHg in eyes continuing on travoprost, and 14.3 ± 1.94 mmHg in eyes switched to TTFC.

**Figure 1:**
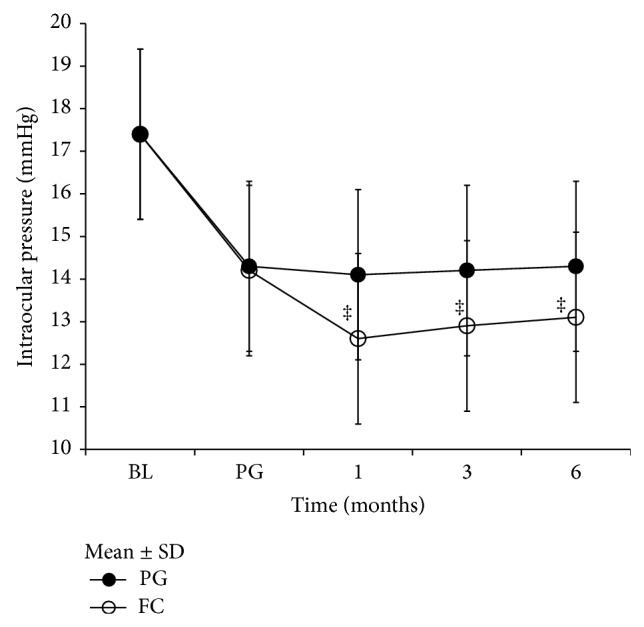


Changes in intraocular pressure following the transition from PG monotherapy to fixed combination therapy are shown in Figure 1. After switching to fixed combination therapy, the mean intraocular pressure was 12.6 ± 1.98 mmHg (*P* < 0.001) at 1 month, 12.9 ± 2.08 mmHg (*P* < 0.001) at 3 months, and 13.1 ± 1.79 mmHg (*P* < 0.001) at 6 months; all values were significantly lower than those observed in eyes continuing on PG. In the PG eyes, the percentage change from BL was −17.82% before switching therapy, −18.97% at 1 month after switching therapy, −18.39% at 3 months, and −17.82% at 6 months. In PG/b eyes, the percentage change from BL was −18.39% before switching therapy, −27.59% at 1 month after switching therapy, −25.86% at 3 months, and −24.71% at 6 months.

**Figure 2:**
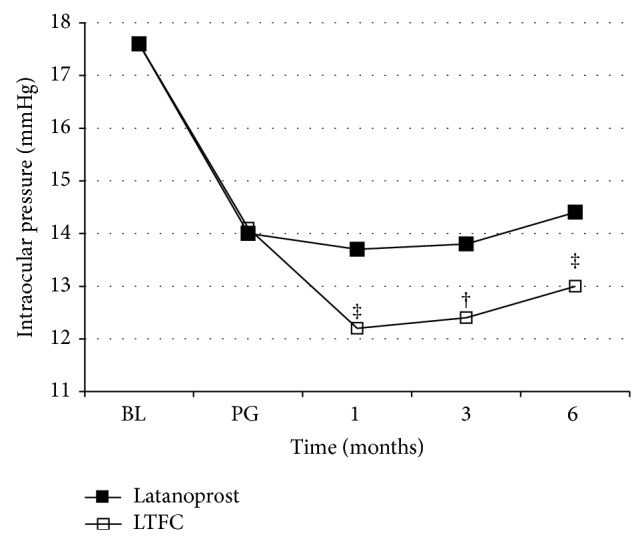


**Figure 3:**
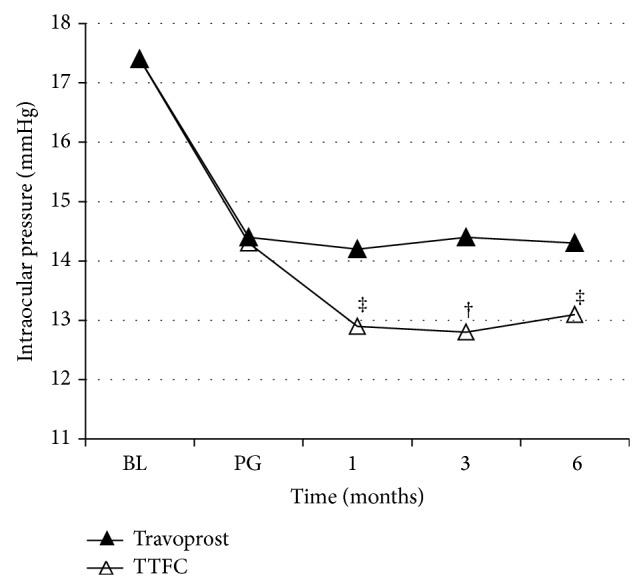


The mean intraocular pressure for each PG medication is shown in Figures 2 and 3. For eyes initially on latanoprost, the mean intraocular pressure was 12.2 ± 1.86 mmHg (*P* = 0.002) 1 month after switching to LTFC, 12.4 ± 1.42 mmHg (*P* = 0.006) at 3 months, and 13.0 ± 1.59 mmHg (*P* = 0.002) at 6 months; all values were significantly lower than those observed in eyes continuing on PG.

For eyes initially on travoprost, the mean intraocular pressure was 12.9 ± 2.00 mmHg (*P* = 0.002) 1 month after switching to TTFC, 12.8 ± 2.70 mmHg (*P* = 0.003) at 3 months, and 13.1 ± 1.94 mmHg (*P* = 0.006) at 6 months; all values were significantly lower than those observed in eyes continuing on PG.

**Figure 4:**
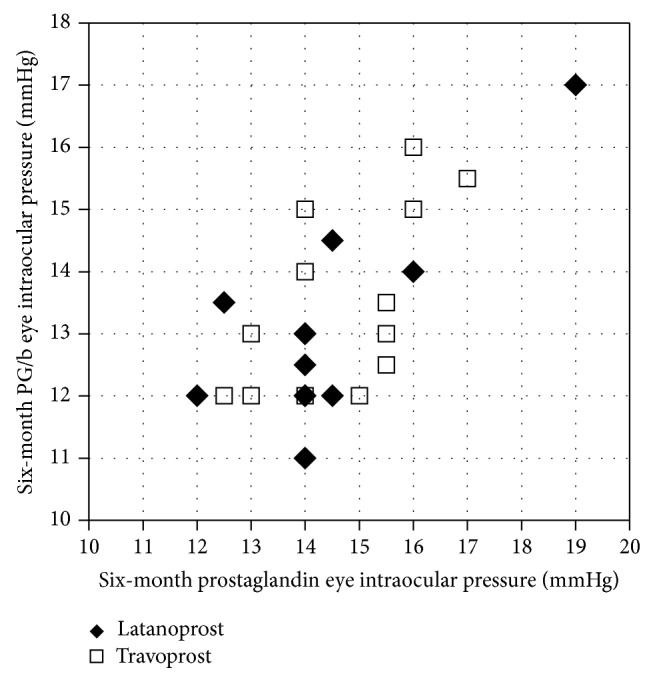


Figure 4 illustrates intraocular pressure differences between the left and right eyes 6 months after switching to fixed-combination therapy. Overall, the intraocular pressure was lower in PG/b eyes from the low to high intraocular pressure zone. However, in 6 eyes, there was no intraocular pressure difference after switching to fixed combination therapy between the left and right eyes. In addition, 2 eyes had high intraocular pressure after switching to PG/b.

**Figure 5:**
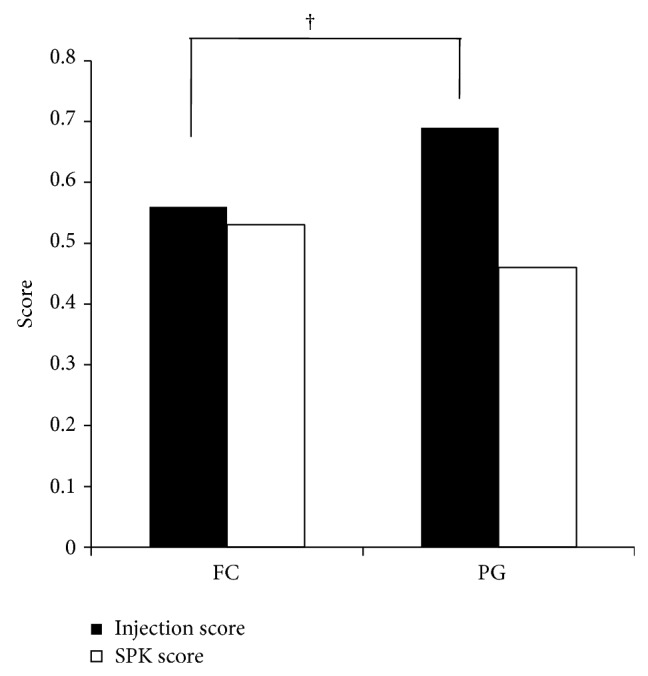


**Figure 6:**
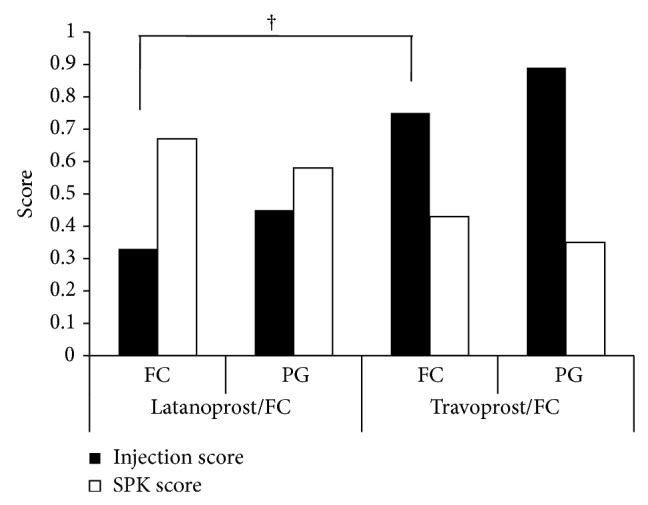


Conjunctival injection and SPK scores 6 months after switching to fixed combination therapy are shown in Figure 5. Overall, the mean injection score was 0.69 for eyes on PG monotherapy and 0.56 for eyes on fixed combination therapy (*P* = 0.028). The mean SPK scores were 0.46 and 0.53, respectively; although the fixed combination therapy SPK score was higher, this difference was not statistically significant (*P* = 0.463). When the scores were analyzed by the type of PG (Figure 6), the conjunctival injection scores for LTFC and latanoprost monotherapy were 0.33 and 0.45, respectively. The conjunctival injection scores for TTFC and travoprost monotherapy were 0.75 and 0.89, respectively. In both groups, the conjunctival injection scores were lower with fixed combination therapy than with PG alone. The SPK scores for LTFC and latanoprost monotherapy were 0.67 and 0.58, respectively (*P* = 0.72). The SPK scores for TTFC and travoprost monotherapy were 0.43 and 0.35, respectively (*P* = 0.71). Thus, in both groups, the SPK scores were higher in subjects on fixed combination therapy than in those receiving PG. The SPK score was higher with LTFC compared to TTFC but no significance (*P* = 0.30). The conjunctival injection score was significantly higher with TTFC compared to LTFC (*P* = 0.005).

## 4. Discussion

In the present study of NTG patients, PG monotherapy was continued in one eye and while the contralateral eye was switched to PG/b fixed combination therapy. Intraocular pressure was significantly lower in the eye switched to fixed combination therapy.

In previous reports, pretherapy intraocular pressure decreased by approximately 25% in patients with high intraocular pressure (≥21 mmHg) treated with PG monotherapy; in patient with intraocular pressure <21 mmHg, intraocular pressure decreased by approximately 14-15% with latanoprost [9–11] and approximately 16% with Travatanz [12, 13]. In combination with timolol, one report stated that the addition of timolol to latanoprost therapy in NTG reduced intraocular pressure by 24% [14]. In the present study, PG monotherapy and fixed combination therapy reduced intraocular pressure by 17% and 24%, respectively; thus, both therapies achieved equal intraocular pressure reductions in NTG patients. Most studies measuring the effect of fixed combination PG/b on intraocular pressure are performed in patients with high intraocular pressure before any therapy. Although direct comparisons to the present study are difficult, studies involving patients with a mean pretherapy pressure of 22–24 mmHg have reported a 31–34% decrease in intraocular pressure with LTFC [15, 16] and a 31-32% decrease with TTFC [17, 18].

According to the Japanese Glaucoma Diagnosis Guidelines, the goal of NTG therapy is to reduce intraocular pressure by 20% from BL or, in severe cases, 30%. In the large CNTGS and EMGT cohort studies, progression of the visual field defect was significantly inhibited when the intraocular pressure was reduced by 25–30% [19, 20].

It is difficult to achieve a 30% decrease in intraocular pressure with PG monotherapy, but the combination of a PG and a beta-blocker can approach a 30% reduction. PG/b fixed combination therapy also achieves the target intraocular pressure for inhibiting progression of the visual field defect.

There were 6 patients with no change in intraocular pressure and 2 patients with increased intraocular pressure after switching to fixed combination therapy. We consider this result may have been occurred from timolol non-respondence or its variation of IOP, or LTFC has stronger feeling of stimulation of eye compared to latanoprost, so stimulation of lacrimal grand reduced of effects of medication. We also consider fixed-comlex medication con not produce the effect of two different medications completely; however, statistical analysis is difficult due to the small number of cases. Further studies are necessary.

Six months after switching to fixed combination therapy, SPK scores were higher in eyes on latanoprost, while conjunctival injection was generally more severe in eyes on travoprost. The higher SPK score in latanoprost compared to travoprost was thought to be that difference of preservative agents. Fixed combinations of both PGs were associated with higher SPK scores and lower conjunctival injection scores. Mechanistically, the beta-blocker effect of timolol may have increased the SPK score by reducing lacrimal secretions and hypoesthesia caused by irritation. Furthermore, timolol likely attenuated conjunctival injection due to its vasoconstrictive effect. Preservatives, particularly benzalkonium chloride (BAK), are thought to play a major role in the development of corneal epithelial disorders. There are topical agents available that contain preservatives other than BAK, have lower concentrations of preservatives, or include a special filter in the eyedropper tip. For example, travoprost/timolol fixed combination eye drops can effectively reduce intraocular pressure with a lower risk of corneal disorder [21, 22] in Japanese patients with primary open-angle glaucoma (POAG) and NTG [7, 23].

In the present study, switching from PG monotherapy to PG/b fixed combination therapy for NTG resulted in a greater intraocular pressure reduction than PG alone without increasing the number of instillations, thereby maintaining patient adherence. The ability to reduce intraocular pressure by approximately 25% from baseline, similar to the effect of monotherapy, with only one instillation per day is considered effective NTG therapy. Proactive use of fixed combination therapy at an early stage is recommended in patients where intraocular pressure treatment is prioritized. Although a 30% reduction in intraocular pressure can be achieved with PG monotherapy, switching to PG/b fixed combination therapy can achieve this 30% reduction more safely, particularly in young patients. However, caution is needed when prescribing fixed combination therapy to elderly patients due to the potential for systemic adverse effects associated with beta-blockers. Therefore, for elderly patients, PG monotherapy is preferred, and PG/b is considered only if necessary.

The subjects in the present study were monitored for 6 months. However, further examination with more patients and a longer follow-up period may be required to fully assess the safety and efficacy of switching from monotherapy to fixed combination therapy.

## Figures and Tables

**Table 1:** Participant demographic data.

	Latanoprost/LTFC		Travoprost/TTFC	
Case number	13		14	
Gender (male/female)	7/6		8/6	
Age	64.0 ± 6.5		53.4 ± 7.6	
Baseline IOP with GAT (mmHg)	17.6 ± 1.35	17.6 ± 1.21	17.4 ± 2.00	17.4 ± 1.86

	Latanoprost	LTFC	Travoprost	TTFC

Spherical equivalent (diopter)	−4.88 ± 2.5	−5.00 ± 3.2	−6.69 ± 4.07	−6.89 ± 4.03
MD score (HFA30/24-2)	−3.91 ± 4.13	−9.34 ± 5.06	−3.51 ± 4.27	−7.12 ± 3.24

IOP: intraocular pressure.

GAT: goldmann applanation tonometer.

LTFC: latanoprost timolol fixed complex.

TTFC: Travoprost timolol fixed complex.
